# Biomechanical Characteristics of Vertical Jumping of Preschool Children in China Based on Motion Capture and Simulation Modeling

**DOI:** 10.3390/s21248376

**Published:** 2021-12-15

**Authors:** Panchao Zhao, Zhongqiu Ji, Ruixiang Wen, Jiahui Li, Xiao Liang, Guiping Jiang

**Affiliations:** College of P.E. and Sports, Beijing Normal University, Beijing 100875, China; jizhongqiu61@bnu.edu.cn (Z.J.); 201731070010@mail.bnu.edu.cn (R.W.); 201821070031@mail.bnu.edu.cn (J.L.); 201921070032@mail.bnu.edu.cn (X.L.); jiang_guiping0401@126.com (G.J.)

**Keywords:** biomechanical, preschool children, vertical jumping, motion capture, simulation modeling

## Abstract

Vertical jumping is one of the basic motor skills, and it is an essential part of many sports. The main purpose of this paper is to investigate characteristics of vertical jumping of children. This paper uses a motion capture system, three-dimensional platforms, and a simulation modeling system to analyze the kinematics and dynamics performance of children’s vertical jumping. The compression time increases from 3 to 4 years old, and flight height and time increases with age and stage gradually. In the compression phase and pushing phase, the hip and knee joint play a major role; in the landing phase, the knee and ankle joint play a major role. Muscle forces are mainly affected by age, and the three types of muscle force had two different trends. The muscle force of the shank and thigh increased with age, and the pelvic girdle muscles showed an “low–high–low” trend. The regression model suggests that the force of GMiP and the hip angular velocity have a great influence on jumping ability. Therefore, if we want to improve the jumping ability of preschool children, we should pay more attention to hip exercises. We should integrate the hip exercises into interesting games, which are more in line with their physical and mental health.

## 1. Introduction

Vertical jumping is a typical stretch-shortening cycle movement (SSC), including preparatory lengthening and rapid shortening of the muscle–tendon unit (MTU) [1]. It is an essential part of many sports, such as basketball, volleyball, and gymnastics, and the performance of this motion directly affects the results of sporting events [2]. In the pyramid model of motor development proposed by Seefeldt [3], vertical jumping is located at the bottom of the pyramid and is the cornerstone of advanced motor skills.

In the preschool period, children’s brains grow rapidly, their perception ability improves rapidly, and their neuromuscular system is developing gradually [4]. Therefore, this stage cannot be analyzed as one age group as a whole but should be subdivided into an age group every one year. The period from 3 to 6 years old is a key period for the development of basic motor skills; if children do not master basic motor skills in this period, the ability to learn complex skills in adulthood will be reduced greatly [5,6]. Some researchers also pay attention to the gender differences of vertical jumping, but studies show that there are no gender differences in this age group [7,8].

At present, studies of vertical jumping focus on the following aspects: neuromuscular coordination, lower limb stiffness, joint dynamics, arm swing, and age characteristics. A research show adults have more feedforward muscle activity than boys when jumping on a single leg; the utilization efficiency of the stretch reflex and elastic potential energy is low in preadolescent children [9]. As age increases, the reflex response and joint–muscle stiffness of lower limb increase [10], and muscle co-contraction decreases [11]. Jumping patterns are different among children of different ages. In order to identify the characteristics of children’s development, data from children are often compared with data from adults. Raffalt et al. [12] found children had more intra-subject variability in the intensity of their muscle activity, which indicated that muscle activity patterns of children are inconsistent, and considerable eccentric muscle contraction was involved in the pushing and landing phase, which limited the muscle activity. The performance of vertical jumping reflects the comprehensive development level of the whole body, but the neglect of longitudinal research of vertical jumping in preschool is shocking.

In the motor developmental field, researchers often use the motor development sequence to observe the development degree of basic movements, which is a qualitative research method; it describes the general characteristics and behavior patterns of children’s movements, which is a fast method for classifying motor skill patterns appearing in the same stage. It can help teachers, parents, and researchers understand the motor development level, notice bottlenecks and key stages, and promote the healthy development of children’s motor abilities [13,14]. At present, the research on vertical jumping pays more attention to the differences between different ages [15,16] but ignores the research on the sequence of motor development.

In summary, this paper uses a motion capture system, two three-dimensional platforms, and a simulation modeling system to analyze the kinematics and dynamics performance of children’s vertical jumping, and this study explore the different characteristics of children’s vertical jumping at different ages and development stages. This paper features two innovations. Firstly, this is the first study to observe vertical jumping in relation to the characteristics of the age and developmental stages in preschool children. Secondly, an inverse dynamics simulation model analyzed the dynamic characteristics of children’s lower limbs, and the 31 muscles’ force of the lower limbs was accurately measured. The research hypotheses were as follows: (1) The characteristics of vertical jumping are different at different ages and development stages. (2) The ability of vertical jumping is mainly reflected in joint kinematics and muscle dynamics.

## 2. Material and Methods

### 2.1. Participants

Ninety preschool children (45 boys and 45 girls) were randomly recruited from a public kindergarten in Beijing. They were divided into a 3-year-old group (3 years ≤ age < 4 years), a 4-year-old group (4 years ≤ age < 5 years), and a 5-year-old group (5 years ≤ age < 6 years). The inclusion criteria required typically developing children who understood the instructions and had good health and normal exercise ability. The exclusion criteria were physical development disorders, cognitive dysfunction diseases, skeletal muscle coordination diseases, etc. Prior to the experiment, the parents of subjects signed informed consent. The study was conducted in accordance with the Declaration of Helsinki, and the protocol was approved by the Ethics Committee of Psychology Department of Beijing Normal University (No. 201910210061). Basic information about the participants is shown in Table 1.

### 2.2. Apparatus and Procedures

#### 2.2.1. Experimental Preparation

**Figure 1:**
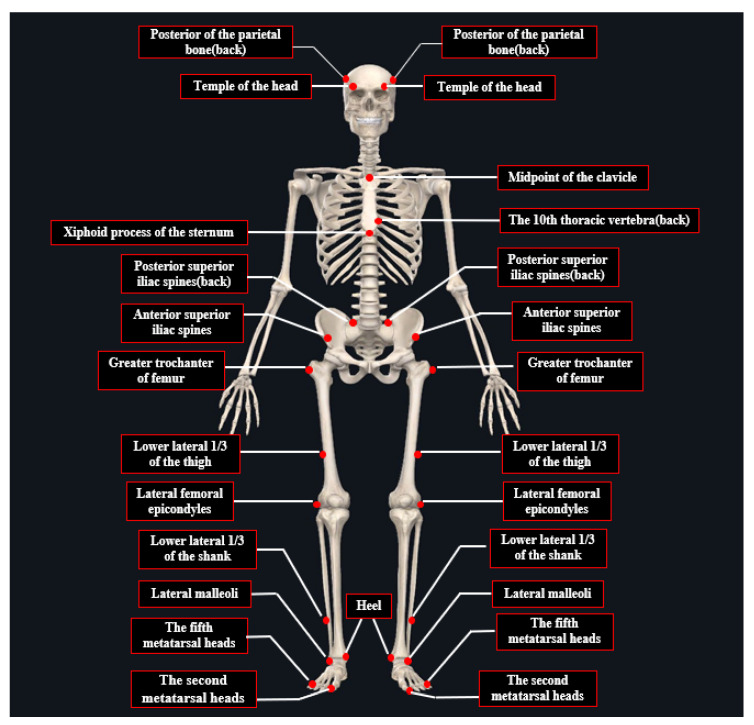
The anatomic location of the plug-in-gait lower limb model.

The subjects dressed in tight test clothing and pasted markers on their body joints according to the Plug-in-Gait lower limb model. Twenty-seven reflective markers were placed at the following anatomic location (Figure 1).

Prior to the test, morphological measurements were performed, such as height, weight, head length, thigh length, shank length, spine length, foot length pelvic width, knee width, and ankle width; these indicators were measured for simulation modeling. Participants warmed up for five minutes; then, subjects stood barefoot with one foot on a platform and another foot on the other platform. They jumped vertically in a way they found most comfortable. The subjects were required to perform in situ vertical jumping with their maximum strength three times, rest for 1 min after each jump, and jump again after the subjects adjust their state. After the test, the tester filters the data, selecting the data for which the capture of markers is the most completed. The kinematic and kinetic data were synchronized in the BTS motion capture software.

#### 2.2.2. Data Acquisition

Kinematic data were collected using an eight-camera BTS motion capture system (SMART DX 700, Bioengineering Technology and Systems, Milano, Italy) sampling at 100 Hz, which is used as action recognition. Kinetic data were collected by two force plates (KISTLER company, kistler928E, Winterthur, Switzerland) sampling at 250 Hz, measured 0.6 × 0.4 × 0.2 m.

#### 2.2.3. Data Processing

After the test, the vertical jumps were classified according to the developmental stages defined by Callahue [17]. The characteristics of motor development in each stage are as follows: (1) In the initial stage, the preparation position and crouch position are inconsistent, the body does not extend when taking off, the child lacks the ability to take off with both feet, and the height of the jump is very limited. (2) In the primary stage, the crouching angle of the knee joint is more than 90 degrees, the child takes off with both feet, the body is not fully extended, and the arms begin to assist in exertion and balance, lacking balance when landing. (3) In the mature stage, the crouching angle of the knee is between 60 and 90 degrees, the whole body is fully extended at takeoff, and the landing is controlled.

**Figure 2:**
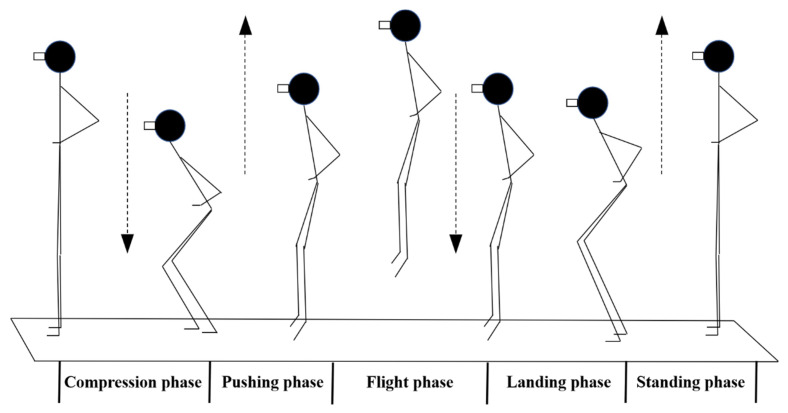
The phase of vertical jumping.

In this paper, vertical jumping was divided into five periods: (1) the compression phase: from upright to the maximum angle of a knee squat; (2) the pushing phase: from the maximum angle of a knee squat to feet off the ground; (3) the flight phase: from feet off the ground to feet on the ground; (4) the landing phase: from feet on the ground to the maximum angle of a knee squat; And (5) the standing phase: from the maximum angle of a knee squat to upright (Figure 2).

Marker trajectories were smoothed using a Butterworth filter with a 10 Hz cut-off, while kinetic data were filtered with a 20 Hz low-pass filter in SMART Analyzer software, the software can calculate spatiotemporal parameters and joint kinematic. The definition of kinematic and dynamic parameters in this paper are seen in Table 2.

#### 2.2.4. Anybody Simulation Operation

The markers trajectory as a C3d format by SMART Tracker software and imported was exported into AnyBody7.0 (Anybody Technology, Aalborg, Denmark) software for simulation modeling analysis. Firstly, morphological measurements indicators of the subjects were input to the script file to establish a personalized simulation model for each subject. Secondly, the kinematic calculation was carried, and then the inverse dynamics calculation was conducted. The muscle force of the right lower limb of each subject was divided by their respective body weight for standardization, expressed by (N/BW).

The human body has a large number of muscles and is therefore prone to the problem of muscle redundancy, in which the number of muscles contained in the system is far greater than the number of muscles needed to balance the load. Muscle recruitment for inverse dynamics in simulation can be used to determine which muscles are involved in maintaining an external load in equilibrium. The optimization process of the skeletal muscle system in the AnyBody Modeling system is as follows [18,19,20]:

Objective function:

(1) $$G(f_{i}{}^{\left(M\right)})$$

Constraint condition:

(2) Cf=d

(3) $$\geq \;0,i\in \left\{1,2\cdots n^{\left(M\right)}\right\}$$

where *G* is the assumed distribution strategy of the central nervous system to the muscle force, *C* is the coefficient matrix of the equation, ƒ is the vector sum of unknown joint and muscle forces, and *D* is the vector sum of known external and inertial forces. $f_{i}{}^{\left(M\right)}$ represents the force of the *i*-th muscle; since the muscle can bear only tensile force, the value is ≥0.

The form of muscle recruitment can be expressed by a polynomial:

(4) $$G=\underset{i}{\sum }{\left(\frac{f_{i}{}^{\left(M\right)}}{N_{i}}\right)}^{p},p\geq 1$$

*N_i_* is the current tensile strength of each muscle, and p is a polynomial power function that can be set to different values according to different situations. The larger the power series, the greater the number of muscles involved in the equilibrium load, and the more significant the synergy is; the maximum synergistic effect occurs when all muscles work together. At this time, the maximum force of any muscle is as small as possible relative to the external load, which is the lowest physiological fatigue standard. The criteria for this recruitment method are as follows:

(5) $$G=max\left(\frac{f_{i}{}^{\left(M\right)}}{N_{i}}\right),i\in \left\{1,2\cdots n^{\left(M\right)}\right\}$$

### 2.3. Statistical Analysis

Descriptive statistics (mean ± standard deviation) were calculated for all data. Two-way ANOVAs were performed to examine the main effect of stage, the main effect of age, and the interaction effects of stage and age. The dependent variables are kinematic and dynamic parameters, and the independent variables are stage and age. The differences between groups were tested by *LSD* method. Linear regression was used to predict the parameters affecting jump ability, and the *Stepwise* method was used for statistical analysis. The dependent variable is flight height, and the independent variables are kinematic parameters during the compression phase, pushing phase, and muscle forces of the lower limb. SPSS 28.0 was used to analyze all data, and *p* < 0.05 indicated significant differences.

## 3. Results

### 3.1. Overall Development Characteristics

**Figure 3:**
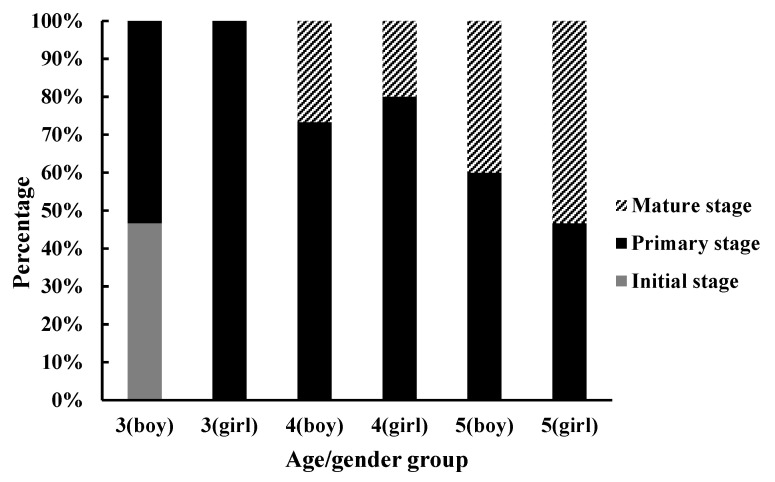
Proportion chart of motor development stages of children in different ages.

Only 3-year-old boys have an initial development stage. Three-year-old girls are all in the primary stage. After 4 years (including 4 years old), the initial stage disappears, and the mature stage appears. Five-year-old children have maximum proportion in the mature stage (Figure 3).

### 3.2. Kinematic Characteristics of Vertical Jumping in Children

#### 3.2.1. Spatiotemporal Parameters of Vertical Jumping

As shown in Table 3, significant differences occurred in compression time, flight time, and flight height.

**Figure 4:**
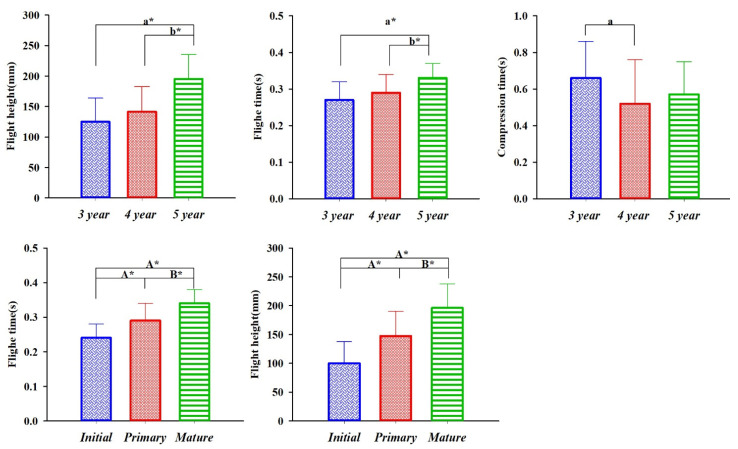
Results of main effect of spatiotemporal parameters. Notes: “a” means a significant difference compared with the 3-year-old group, “a*” means a very significant difference. “b*” means a very significant difference. A* means a very significant difference compared with the initial stage; B* means a very significant difference compared with the primary stage.

AS shown in Figure 4, flight time and flight height have the main effects of stage and age and tend to increase gradually. Compression time has the main effect of age (*p* < 0.05).

#### 3.2.2. Joint Angle and Angular Velocity Parameters of Vertical Jumping

As shown in Table 4, significant differences occurred in hip max flexion angle, spine max tilt angle, hip ROM in compression phase, knee ROM in pushing phase, knee ROM in landing phase, ankle ROM in landing phase, and hip max angular velocity in pushing phase.

As shown in Table 5 and Table 6, hip max flexion angle, spine max tilt angle, hip ROM in compression phase, knee ROM in pushing phase, knee ROM in landing phase, and ankle ROM (deg)-landing phase increase with stage. Ankle ROM in landing phase and hip max angular velocity in pushing phase increase with age.

### 3.3. Peak Muscle Force of Vertical Jumping in Children

As shown in Table 7, significant differences occurred in the following muscle: Soleus Medialis, Soleus Lateralis, Gastrocnemius Lateralis, Vastus Lateralis Inferior, Vastus Lateralis Superior, Vastus Medialis Mid, Vastus Medialis Superior, Vastus Intermedius, Rectus Femoris, Piriformis, Adductor Magnus Distal, Gemellus Inferior, Gemellus Superior, Obturator Internus, Poplitues, and Quadratus Femoris.

As shown in Table 8 and Table 9, the muscle forces of SM, SL, GL, VLS, VMM, VMS, VI, RF, Pir, AMD, GI, GS, OI, Popliteus, and QF increase with age. The muscle forces of VLI and AMD increase with stage.

### 3.4. Influencing Factors of Jumping Ability of Preschool Children

Linear regression was used to predict the parameters affecting jump ability. Data display that ankle ROM in pushing phase, spine max tilt angle, hip max angular velocity, muscle force of GMiP, and muscle force of GM were incorporated into the model. The final regression equation is:

Y = −85.527 + 1.243 ∗ ankle ROM + 0.754 ∗ spine max tilt angle + 9.354 ∗ hip max angular velocity + 33.847 ∗ GMiP + 2.640 ∗ GM

The higher the number before the indicator, the greater the influence on the dependent variable. VIF is the variance expansion coefficient; when the value is less than 3, it indicates that there is no collinearity problem; when Durbin–Watson’s value is between 1.5–2.5, it indicates that there is no self-correlation between samples, and the values of VIF and DW are within a reasonable range. R^2^ indicates that the regression model could explain 64.1% of the variance of the dependent variable (Table 10).

## 4. Discussion

The main purpose of this paper is to investigate characteristics of vertical jumping of children. The spatial and temporal parameters, joint kinematics, and muscle forces of the lower limb were evaluated. As motor development matured and age increased, most of the indicators showed an increasing linear trend, while the muscle force of the pelvic girdle showed an “low–high–low” trend. This paper uses flight height to evaluate ability of children’s vertical jumping. Finally, a regression equation is established, which reflects the influence of different factors on flight height.

In spatiotemporal parameters of vertical jumping, from 3 to 4 years old, the compression time increase, it means the 4year-old children move faster. The flight height and time increases with age and stage gradually. It is related to the increase of lower-limb muscle force, which is consistent with previous studies [21]. Joint kinematics data show that the mature stage has greater joint motion, and the differences are mainly reflected in the compression phase, pushing phase, and stability phase. In the compression phase and pushing phase, the hip and knee joint play a major role, and they work together to promote the take-off. In the landing phase, the knee and ankle joint play a major role, and they work together to ensure a stable landing. Many studies have proposed that the knee is the most important joint in generating energy for jumping [22,23]. Harrison et al. [24] observed that more movement of knee and effective use of knee extensor are the signs of a mature vertical jump mode. In the compression phase, adults produce more energy from the knee, but the hip and knee are equally important during the pushing phase, and the utilization rate of the hip is significantly lower in children [25]. With the increase of age, the motion parameters also show an increasing trend, mainly reflected in the hip angular velocity in pushing phase and the ankle ROM in landing phase.

Muscle forces are mainly affected by age. The data mainly include three muscle types: (1) muscles in the back of the shank: SM, SL, and GL, which are responsible for the flexion of knee and ankle.;(2) muscles in the front of the thigh: VLI, VMM, VMS, VI, and RF, which are responsible for the flexion of hip; And (3) muscles of the pelvic girdle: Pir, AMD, GI, GS, OI, Pop, and QF, which are responsible for stabilizing the pelvis. In this study, the maximum muscle force was VLS; however, a simulation study on adults found that [26] the gluteal muscle is the most powerful muscle, which reflects the difference between children and adults. Due to their young age and incomplete muscle development, children do not know how to coordinate muscle force. Yahya et al. [27] studied the jumping movement of basketball players, and they found that the main muscle used in jumping was semitendinosus, which was inconsistent with the results of our article; the reason may be that the research objects are different, and the jumping movements involved in basketball are different in this experiment. In this study, the three types of muscle forces have two different trends. The muscle forces of shank and thigh increase with age, and the pelvic girdle muscles show a “low–high–low” trend.

Vertical jumping is evaluated by the height of flight [28]. Relevant studies found that the flight height is affected by age and development stage [6]. However, what motion characteristics affect the flight height? There is no research on this issue. Therefore, this paper verifies which factors have an impact on flight height and the degree of impact. The data suggest that the force of GMiP and the hip angular velocity have a great influence on it. Therefore, if we want to improve the jumping ability of preschool children, we should pay more attention to hip exercise. Children at this age do not adapt to high-intensity sports training [29]; the hip exercise should be integrated into interesting games, which are more in line with their physical and mental health.

Limitations:

There are some limitations to this study. First, this paper does not analyze the gender differences of preschool children. Second, kinematic parameters only study the motion data of sagittal plane but lack the frontal plane and horizontal plane. In future research, the discussion of gender factors should be added, and more comprehensive kinematic data should be added.

## 5. Conclusions

Both developmental stage and age have an impact on the characteristics of vertical jumping of preschool children, but they have no interactive effect. Older children and children in the mature stage have more flexible joints, and the range of motion is greater, so it generates more joint motion. In the compression phase and pushing phase, the hip and knee joint play a major role; in the landing phase, the knee and ankle joint play a major role. The difference of muscle force is reflected in muscles in the back of the shank, muscles in the front of the thigh, and the muscles of the pelvic girdle. Muscle forces of the shank and thigh increase with age, and muscle forces of the pelvic girdle show an “low–high–low” trend. The regression model suggests that the muscle force of GMiP and the hip angular velocity have a great influence on jumping ability. Therefore, if we want to improve the jumping ability of preschool children, we should pay more attention to hip exercise. We should integrate the hip exercise into interesting games, which are more in line with their physical and mental health.

## Figures and Tables

**Table 1:** Basic information about the participants ($\bar{X}$ ± S).

Age	*n*	Height (cm)	Weight (kg)	BMI (kg/m^2^)
3 years old	30	101.34 ± 4.01	16.62 ± 2.00	16.20 ± 1.83
4 years old	30	109.14 ± 4.05 ^a^	18.36 ± 2.47 ^a^	15.36 ± 1.25
5 years old	30	116.36 ± 4.11 ^ab^	22.38 ± 3.95 ^ab^	16.44 ± 2.05

Notes: “a” means a significant difference compared with the 3-year-old group, and “b” means a significant difference compared with the 4-year-old group.

**Table 2:** Kinematic and dynamic parameters in vertical jumping.

Parameters	Definition	Type
Time of five phases	Time span of each movement phase	Spatiotemporal
Squat depth	The vertical downward distance of the midpoint of the posterior superior iliac from upright to the maximum angle of a knee squat during the compression phase	Spatiotemporal
Flight height	The vertical upward distance of the midpoint of the posterior superior iliac from the upright to the highest point of the upward jump	Spatiotemporal
Landing buffer depth	The vertical downward distance of the midpoint of the posterior superior iliac from upright to the maximum angle of a knee squat during the landing phase	Spatiotemporal
Maximum flexion angle of the hip	The maximum angle between the anterior superior iliac spine, greater trochanter of femur, and the lateral femoral epicondyles projected on the sagittal plane	Joint parameters
Maximum flexion angle of the knee	The maximum angle between the greater trochanter of femur, the lateral femoral epicondyles, and the lateral malleoli projected on the sagittal plane	Joint parameters
Maximum flexion angle of the ankle	The maximum angle of the lateral femoral epicondyles, lateral malleoli, and the fifth metatarsal head projected onto the sagittal plane	Joint parameters
Maximum tilt angle of the spine	The max angle between the line between the midpoint of the clavicle and T10 projected onto the sagittal plane and the vertical axis	Joint parameters
Hip range of motion	The maximum flexion angle minus the minimum flexion angle during the compression phase, pushing phase, and landing phase	Joint parameters
Knee range of motion	The maximum flexion angle minus the minimum flexion angle during the compression phase, pushing phase, and landing phase	Joint parameters
Ankle range of motion	The maximum flexion angle minus the minimum flexion angle during the compression phase, pushing phase, and landing phase	Joint parameters
Angular velocity of the hip	Maximum angular velocity of the hip in the pushing phase	Joint parameters
Angular velocity of the knee	Maximum angular velocity of the knee in the pushing phase	Joint parameters
Angular velocity of the ankle	maximum angular velocity of the ankle in the pushing phase	Joint parameters
Muscle force	Maximum muscle force of 31 lower-limb muscles	Dynamic parameters

**Table 3:** Results of main and interaction effect of spatiotemporal parameters.

Dependent Variable	Main/Interaction Effect	F	Sig.	η_partial_^2^
Compression time	Stage	3.103	0.051	0.075
	Age	5.349	0.007	0.122
	Stage × Age	0.124	0.726	0.002
Pushing time	Stage	2.187	0.119	0.051
	Age	1.736	0.183	0.041
	Stage × Age	0.049	0.826	0.001
Flight time	Stage	5.695	0.005	0.122
	Age	5.833	0.004	0.125
	Stage × Age	2.034	0.158	0.024
Landing time	Stage	0.475	0.624	0.011
	Age	1.657	0.197	0.039
	Stage × Age	0.339	0.562	0.004
Standing time	Stage	0.283	0.754	0.007
	Age	1.368	0.260	0.032
	Stage × Age	0.257	0.614	0.003
Total time	Stage	1.765	0.178	0.041
	Age	2.726	0.071	0.062
	Stage × Age	0.037	0.847	0.000
Squat depth	Stage	2.583	0.082	0.059
	Age	0.157	0.855	0.004
	Stage × Age	0.464	0.497	0.006
Flight height	Stage	6.493	0.002	0.137
	Age	10.704	<0.001	0.207
	Stage × Age	0.742	0.392	0.009
Buffer time	Stage	0.942	0.394	0.023
	Age	0.112	0.894	0.003
	Stage × Age	1.118	0.294	0.014

**Table 4:** Results of main and interaction effect of joint angle and angular velocity parameters.

Dependent Variable	Main/Interaction Effect	F	Sig.	η_partial_^2^
Ankle max flexion/extension (deg)	Stage	0.273	0.762	0.007
	Age	0.956	0.389	0.023
	Stage × Age	0.438	0.510	0.005
Knee max flexion/extension (deg)	Stage	2.329	0.104	0.057
	Age	0.081	0.922	0.002
	Stage × Age	0.642	0.425	0.008
Hip max flexion/extension (deg)	Stage	6.644	0.002	0.163
	Age	2.008	0.142	0.056
	Stage × Age	2.603	0.111	0.037
Spine max tilt (deg)	Stage	3.984	0.022	0.092
	Age	0.248	0.781	0.006
	Stage × Age	2.630	0.109	0.032
Knee ROM(deg)-compression phase	Stage	3.159	0.048	0.072
	Age	0.350	0.706	0.009
	Stage × Age	1.557	0.216	0.019
Ankle ROM(deg)-compression phase	Stage	0.256	0.775	0.006
	Age	1.281	0.283	0.030
	Stage × Age	0.201	0.655	0.002
Hip ROM (deg)-compression phase	Stage	3.552	0.033	0.083
	Age	0.568	0.569	0.014
	Stage × Age	1.195	0.278	0.015
Knee ROM (deg)-pushing phase	Stage	3.913	0.024	0.088
	Age	0.112	0.894	0.003
	Stage × Age	0.156	0.694	0.002
Ankle ROM (deg)-pushing phase	Stage	2.331	0.104	0.054
	Age	0.647	0.526	0.016
	Stage × Age	0.569	0.453	0.007
Hip ROM (deg)-pushing phase	Stage	3.053	0.053	0.070
	Age	0.807	0.450	0.020
	Stage × Age	0.019	0.890	0.000
Knee ROM (deg)-landing phase	Stage	3.771	0.027	0.085
	Age	0.225	0.799	0.006
	Stage × Age	0.458	0.501	0.006
Ankle ROM (deg)-landing phase	Stage	1.543	0.220	0.036
	Age	4.556	0.013	0.100
	Stage × Age	0.935	0.336	0.011
Hip ROM (deg)-landing phase	Stage	1.021	0.365	0.024
	Age	0.054	0.947	0.001
	Stage × Age	0.867	0.355	0.010
Knee max angular velocity(rad/s)-pushing phase	Stage	1.104	0.336	0.026
	Age	1.829	0.167	0.043
	Stage × Age	0.243	0.623	0.003
Ankle max angular velocity(rad/s)-pushing phase	Stage	0.103	0.902	0.003
	Age	1.331	0.270	0.031
	Stage × Age	0.677	0.413	0.008
Hip max angular velocity(rad/s)-pushing phase	Stage	1.314	0.274	0.031
	Age	4.113	0.020	0.091
	Stage × Age	.238	0.627	0.003

**Table 5:** Results of stage effect of joint angle.

Stage	Initial Stage	Primary Stage	Mature Stage
Hip max flexion/extension (deg)	84.042 ± 10.909	71.672 ± 3.814	50.902 ± 6.781 ^AB^
Spine max tilt (deg)	28.450 ± 5.591	33.693 ± 1.803	44.223 ± 3.210 ^AB^
Hip ROM (deg)-compression phase	77.184 ± 10.413	79.291 ± 3.647	97.198 ± 6.458 ^B^
Knee ROM (deg)-pushing phase	51.971 ± 7.514	55.756 ± 2.617	70.315 ± 4.601 ^AB^
Knee ROM (deg)-landing phase	21.236 ± 3.942	28.077 ± 1.373	35.408 ± 2.414 ^B^
Ankle ROM (deg)-landing phase	23.833 ± 3.523	34.001 ± 1.218 ^A^	39.317 ± 2.157 ^A*B^
Hip max angular velocity(rad/s)-pushing phase	7.526 ± 0.900	9.395 ± 0.311	10.463 ± 0.551 ^A^

Notes: A means a significant difference compared with the initial stage, A* means a very significant difference. B means a significant difference compared with the primary stage.

**Table 6:** Results of age effect of joint angle.

Age	3 Years Old	4 Years Old	5 Years Old
Ankle ROM (deg)-landing phase	26.573 ± 2.034	36.027 ± 2.012 ^a^	39.634 ± 1.706 ^a*^
Hip max angular velocity(rad/s)-pushing phase	8.140 ± 0.0520	9.232 ± 0.514	10.946 ± 0.436 ^a*b^

Notes: “a” means a significant difference compared with the 3-year-old group, “a*” means a very significant difference. “b” means a significant difference compared with the 4-year-old group.

**Table 7:** Results of main and interaction effect of muscle force parameters.

Dependent Variable	Main/Interaction Effect	F	Sig.	η_partial_^2^
Soleus Medialis (SM)	Stage	2.582	0.082	0.058
	Age	19.322	<0.001	0.315
	Stage × Age	1.952	0.166	0.023
Soleus Lateralis (SL)	Stage	0.726	0.487	0.017
	Age	9.135	<0.001	0.179
	Stage × Age	0.719	0.399	0.008
Gastrocnemius Lateralis (GL)	Stage	0.081	0.923	0.002
	Age	6.171	0.003	0.128
	Stage × Age	0.403	0.527	0.005
Gastrocnemius Medialis (GM)	Stage	0.982	0.379	0.023
	Age	2.878	0.062	0.064
	Stage × Age	0.000	0.992	0.000
Peroneus Brevis (PB)	Stage	1.247	0.293	0.029
	Age	2.922	0.059	0.065
	Stage × Age	0.111	0.740	0.001
Peroneus Longus (PL)	Stage	0.590	0.557	0.014
	Age	1.791	0.173	0.041
	Stage × Age	0.835	0.363	0.010
Vastus Lateralis Inferior (VLI)	Stage	3.407	0.038	0.075
	Age	3.042	0.053	0.068
	Stage × Age	2.254	0.137	0.026
Vastus Lateralis Superior (VLS)	Stage	0.788	0.458	0.018
	Age	13.694	<0.001	0.246
	Stage × Age	0.007	0.934	0.000
Vastus Medialis Inferior (VMI)	Stage	1.943	0.150	0.044
	Age	3.177	0.047	0.070
	Stage × Age	0.863	0.356	0.010
Vastus Medialis Mid (VMM)	Stage	1.254	0.291	0.029
	Age	11.076	<0.001	0.209
	Stage × Age	0.002	0.965	0.000
Vastus Medialis Superior (VMS)	Stage	2.217	0.115	0.050
	Age	6.475	0.002	0.134
	Stage × Age	1.847	0.178	0.022
Vastus Intermedius (VI)	Stage	0.906	0.408	0.021
	Age	10.510	<0.001	0.200
	Stage × Age	0.010	0.921	0.000
Rectus Femoris (RF)	Stage	1.174	0.314	0.027
	Age	10.459	<0.001	0.199
	Stage × Age	1.946	0.167	0.023
Semitendinosus (Sd)	Stage	0.707	0.496	0.017
	Age	2.821	0.065	0.063
	Stage × Age	0.117	0.733	0.001
Semimembranosus (Sb)	Stage	1.755	0.179	0.040
	Age	0.654	0.522	0.015
	Stage × Age	1.247	0.267	0.015
Biceps Femoris CaputLongum (BFCL)	Stage	0.896	0.412	0.021
	Age	1.263	0.288	0.029
	Stage × Age	0.274	0.602	0.003
Gluteus Minimus Anterior (GMiA)	Stage	0.162	0.850	0.004
	Age	0.703	0.498	0.016
	Stage × Age	0.541	0.464	0.006
Gluteus Minimus Mid (GMiM)	Stage	0.637	0.531	0.015
	Age	0.181	0.835	0.004
	Stage × Age	0.000	0.983	0.000
Gluteus Minimus Posterior (GMiP)	Stage	0.553	0.577	0.013
	Age	2.541	0.085	0.057
	Stage × Age	1.201	0.276	0.014
Gluteus Medius Anterior (GMeA)	Stage	1.039	0.358	0.024
	Age	0.466	0.629	0.011
	Stage × Age	0.121	0.728	0.001
Gluteus Medius Posterior (GMeP)	Stage	0.295	0.745	0.007
	Age	2.952	0.058	0.066
	Stage × Age	0.511	0.477	0.006
Gluteus Maximus Superior (GMaS)	Stage	0.814	0.446	0.019
	Age	2.323	0.104	0.052
	Stage × Age	4.054	0.047	0.046
Gluteus Maximus Inferior (GMaI)	Stage	1.527	0.223	0.035
	Age	2.616	0.079	0.059
	Stage × Age	1.946	0.167	0.023
Piriformis (Pir)	Stage	2.156	0.122	0.049
	Age	4.703	0.012	0.101
	Stage × Age	0.549	0.461	0.006
Adductor Magnus Distal (AMD)	Stage	3.975	0.022	0.086
	Age	4.586	0.013	0.098
	Stage × Age	1.419	0.237	0.017
Adductor Magnus Mid (AMM)	Stage	1.644	0.199	0.038
	Age	1.702	0.189	0.039
	Stage × Age	0.393	0.532	0.005
Gemellus Inferior (GI)	Stage	0.407	0.667	0.010
	Age	8.714	<0.001	0.172
	Stage × Age	0.118	0.733	0.001
Gemellus Superior (GS)	Stage	0.002	0.998	0.000
	Age	8.935	<0.001	0.175
	Stage × Age	0.018	0.895	0.000
Obturator Internus (OI)	Stage	0.495	0.612	0.012
	Age	11.771	<0.001	0.219
	Stage × Age	0.022	0.883	0.000
Poplitues (Pop)	Stage	2.610	0.079	0.059
	Age	6.969	0.002	0.142
	Stage × Age	1.455	0.231	0.017
Quadratus Femoris (QF)	Stage	1.270	0.286	0.029
	Age	9.616	<0.001	0.186
	Stage × Age	2.150	0.146	0.025

**Table 8:** Results of age effect of muscle force.

Age	3 Years Old	4 Years Old	5 Years Old
SM	4.152 ± 1.508	4.356 ± 1.054	6.716 ± 2.550 ^a*b*^
SL	8.668 ± 2.567	11.968 ± 3.717 ^a*^	13.620 ± 4.14 ^a*^
GL	5.055 ± 1.915	6.965 ± 2.283 ^a*^	7.321 ± 2.387 ^a*^
VLS	14.469 ± 5.507	14.347 ± 3.717	22.990 ± 7.667 ^a*b*^
VMM	3.087 ± 1.459	3.534 ± 0.973	5.325 ± 1.792 ^a*b*^
VMS	3.033 ± 1.302	3.524 ± 1.282	4.919 ± 1.576 ^a*b*^
VI	4.502 ± 1.494	4.500 ± 1.153	6.684 ± 2.210 ^a*b*^
RF	3.498 ± 1.057	3.422 ± 1.625	4.330 ± 2.230 ^a*b*^
Pir	1.337 ± 0.804	1.948 ± 1.010 ^a^	1.742 ± 0.786
AMD	3.813 ± 1.129	3.554 ± 1.219	4.866 ± 1.963 ^ab*^
GI	0.459 ± 0.205	0.784 ± 0.365 ^a*^	0.545 ± 0.278 ^b*^
GS	0.366 ± 0.165	0.586 ± 0.187 ^a*^	0.428 ± 0.205 ^b^
OI	2.430 ± 1.063	4.396 ± 1.415 ^a*^	3.242 ± 1.623 ^ab*^
Pop	0.538 ± 0.223	0.574 ± 0.270	0.921 ± 0.368 ^a*b*^
QF	1.231 ± 0.431	2.125 ± 0.790 ^a*^	1.597 ± 0.991 ^b^

**Notes:** “a” means a significant difference compared with the 3-year-old group, “a*” means a very significant difference. “b” means a significant difference compared with the 4-year-old group, “b*” means a very significant difference compared with the 4-year-old group.

**Table 9:** Results of stage effect of muscle force.

Stage	Initial Stage	Primary Stage	Mature Stage
VLI	0.613 ± 0.181	0.739 ± 0.341	1.066 ± 0.394 ^A*B*^
AMD	3.506 ± 1.734	3.778 ± 0.173	5.151 ± 0.166 ^AB^

**Notes:** A means a significant difference compared with the initial stage, A* means a very significant difference. B means a significant difference compared with the primary stage, B* means a very significant difference compared with the primary stage.

**Table 10:** Regression analysis of influencing factors of vertical jumping.

Dependent Variable	Model	Unstandardized Coefficients		Standardized Coefficients			Collinearity		Durbin-Watson	R^2^
		B	Std. Error	Beta	t	Sig.	Tolerance	VIF		
Flight height (mm)	(Constant)	−85.527	24.132		−3.544	<0.001			1.876	0.641
	Ankle ROM(deg)-pushing phase	1.243	0.307	0.319	4.044	<0.001	0.931	1.074		
	Spine max tilt angle(deg)	0.754	0.292	0.212	2.577	0.012	0.852	1.174		
	Hip max angular velocity(rad/s)-pushing phase	9.354	2.007	0.392	4.660	<0.001	0.819	1.221		
	Gluteus Minimus Posterior (GMiP)	33.847	7.657	0.341	4.420	<0.001	0.974	1.027		
	Gastrocnemius Medialis (GM)	2.640	1.203	0.171	2.195	0.032	0.953	1.049		

## Data Availability

The data presented in this study are available on request from the corresponding author.
